# The Utility of the Mini-Clinical Evaluation Exercise (Mini-CEX) in the Emergency Department: A Systematic Review and Meta-Analysis Evaluating the Readability, Feasibility, and Acceptability of Mini-CEX Utilization

**DOI:** 10.7759/cureus.44443

**Published:** 2023-08-31

**Authors:** Hany A Zaki, Yavuz Yigit, Eman Shaban, Ahmed E Shaban, Amr Elmoheen, Khalid Bashir, Kaleem Basharat, Mohamed Ali, Baha Hamdi Alkahlout

**Affiliations:** 1 Emergency Medicine, Hamad Medical Corporation, Doha, QAT; 2 Blizard Institute, Queen Mary University, London, GBR; 3 Cardiology, Al Jufairi Diagnosis and Treatment, Doha, QAT; 4 Internal Medicine, Mansoura General Hospital, Mansoura, EGY; 5 Medicine, Mansoura University Faculty of Medicine, Mansoura, EGY; 6 Medicine, Qatar University, Doha, QAT

**Keywords:** mini-evaluation exercise, satisfaction, feasibility, acceptability, reliability, emergency department, mini-clinical examination, modified mini-cex, mini-clinical evaluation exercise, mini-cex

## Abstract

Assessment tools, such as the mini-clinical evaluation exercise (mini-CEX), have been developed to evaluate the competence of medical trainees during routine duties. However, their effectiveness in busy environments, such as the emergency department (ED), is poorly understood. This study assesses the feasibility, reliability, and acceptability of implementing the mini-CEX in the ED.

PubMed, Google Scholar, ScienceDirect, Scopus, and Web of Science databases were scoured for observational and randomized trials related to our topic. Moreover, a manual search was also conducted to identify additional studies. After the literature search, data were extracted from studies that were eligible for inclusion by two independent reviewers. When applicable, meta-analyses were performed using the Comprehensive Meta-Analysis software. In addition, the methodological quality of studies was evaluated using the Newcastle-Ottawa Scale.

Of the 2,105 articles gathered through database and manual searches, only four met the criteria for inclusion in the review. A combined analysis of three studies revealed that trainee-patient interactions averaged 16.05 minutes (95% CI = 14.21-17.88), and feedback was given in about 10.78 minutes (95% CI = 10.19-11.38). The completion rates for mini-CEX were high: 95.7% (95% CI = 87.6-98.6) for medical trainees and 95.8% (95% CI = 89.7-98.3) for assessors. Satisfaction with mini-CEX was notable, with 63.5% (95% CI = 51.5-74.1) of medical trainees and 75.7% (95% CI = 63.9-84.6) of assessors expressing contentment. Qualitative data from one study demonstrated that 70.6% of faculty members could allocate suitable time for mini-CEX during their clinical shifts.

The mini-CEX is a feasible and acceptable assessment tool within the ED. Furthermore, there is evidence to suggest that it might be reliable.

## Introduction and background

Assessment is a core component of medical training. Therefore, it must be reliable, valid, feasible, acceptable, and beneficial to the learning progress of medical students and residents. Numerous workplace-based assessment (WBA) tools have been developed to evaluate medical trainees’ clinical competence and professional behavior during their routine clinical duties to fulfill this purpose. One of the most frequently used WBAs, generated by the American Board of Internal Medicine, is the mini-clinical evaluation exercise (mini-CEX) [1,2].

The mini-CEX is a valuable assessment tool that can evaluate various clinical skills, including interview skills, physical examination skills, professionalism, clinical judgment skills, counseling skills, organization skills, and overall clinical competence. Research has shown that mini-CEX is successfully adopted in various clinical situations as a formative and summative assessment tool [3-6]. However, its use in the emergency department (ED) is poorly documented. The ED is a hectic medical department due to the emergent nature of the patients being managed there. As a result, structured training programs used in other departments, such as lunchtime conferences and morning reports, are challenging to implement. Therefore, the mini-CEX has been used to overcome these challenges.

Despite the widespread application of the mini-CEX, concerns regarding its validity, feasibility, and acceptability when assessing the clinical performance of medical trainees in the ED have arisen. As a result, we have chosen to conduct this meta-analysis to determine the effectiveness of the mini-CEX in the ED. To evaluate its effectiveness, our objective is to address the following research question: What are the reliability, acceptability, and feasibility of utilizing the mini-CEX to evaluate medical trainees in the ED?

## Review

Methodology

Eligibility Criteria

Two independent reviewers formulated the criteria to include and exclude studies in the present review. The criteria for including articles were as follows: observational and randomized studies published in the English language; studies reporting the utility of computer-based or paper-based mini-CEX in the ED or emergency medicine; studies with empirical data (either quantitative or qualitative) related to reliability, acceptability, and feasibility; and studies with modified and original forms of mini-CEX.

On the other hand, studies were excluded if they met the following criteria: studies that reported additional WBA tools such as objective structured clinical examinations, direct observation of procedural skills, multi-source feedback, and case-based discussion; studies in which mini-CEX was used to assess trainees in other departments or medical settings, e.g., ambulatory, outpatient, or inpatient; studies in which the mini-CEX was combined with other assessment tools; studies designed as either abstracts, systematic reviews, and meta-analyses; or study protocols.

Information Sources and Search Strategy

The detailed search for articles relevant to our topic was performed using two different approaches. First, we searched PubMed, Google Scholar, ScienceDirect, Scopus, and Web of Science databases for all literature published up to July 2023. In this approach, the following MeSH terms and keywords were employed: (“Mini-CEX” OR “Mini-Clinical Evaluation Exercise” OR “modified mini-CEX” OR “Mini evaluation exercise” OR “mini-clinical examination”) AND (“Emergency department” OR “emergency medicine” OR “ED” OR “Emergency room” OR “Emergency physicians”) AND (“Reliability” OR “acceptability” OR “feasibility” OR “satisfaction” OR “feedback time” OR “observation time” OR “clinical competence” OR “clinical skills”). In addition, we performed a manual search by screening the reference lists of articles related to our topic to find additional studies.

Selection Process

Once the database search process was completed, all the records were imported to the Covidence® Systematic Review Software, where all the exact or close duplicates were excluded. The reviewers tasked with the literature search then screened the records based on the titles, abstracts, and full texts. All discrepancies during this process were resolved by consulting a third reviewer.

Data Collection and Data Items

Two independent reviewers were responsible for gathering data from the selected studies. The abstracted data included the following information: author ID (publication year and first author’s surname), study design, study location or country, characteristics of participants (number of trainees and raters in the sample), study duration, and the outcomes assessed in each study. Any inconsistencies during this phase were resolved through constructive discussions between the two reviewers or by seeking input from a third reviewer if consensus could not be reached.

Outcome Measures and Definitions

The primary outcomes measured in our study were reliability, feasibility, and acceptability. Feasibility was assessed by evaluating the observation time, feedback time, and completion rate of mini-CEX assessment forms. On the other hand, acceptability was defined as the assessor and trainee’s satisfaction with the mini-CEX as an assessment and formative tool.

Quality Assessment

All studies in this systematic review were of an observational nature, and the quality assessment utilized the Newcastle-Ottawa Scale (NOS). This tool involved an experienced reviewer evaluating articles across three domains, namely, selection, comparability, and outcomes. Within the selection domain, the assessment covered the representativeness of the exposed cohort, the selection of the non-exposed group, exposure ascertainment, and the presence of initial study outcomes. The comparability domain evaluated whether confounders were adjusted for in the statistical analysis, while the outcome domain focused on follow-up duration adequacy, outcome ascertainment, and sufficient follow-up duration. A star was assigned to each fully addressed criterion; otherwise, no star was given. Furthermore, the overall methodological quality of each study was assessed by converting NOS scores to the standards set by the Agency for Healthcare Research and Quality.

Data Synthesis

The acceptability, reliability, and feasibility of the data were analyzed using meta-analyses with Comprehensive Meta-Analysis Software. To account for heterogeneity, a random-effect model was used to provide conservative pooled effect sizes. For continuous outcome data, mean, and SDs were pooled, while the event rate was used for dichotomous data. If SD was not provided, online calculators were used, and the ranges were divided by four for smaller sample sizes and by six for larger ones. Additionally, a 95% CI was utilized during statistical analyses.

Results

Study Selection

**Figure 1:**
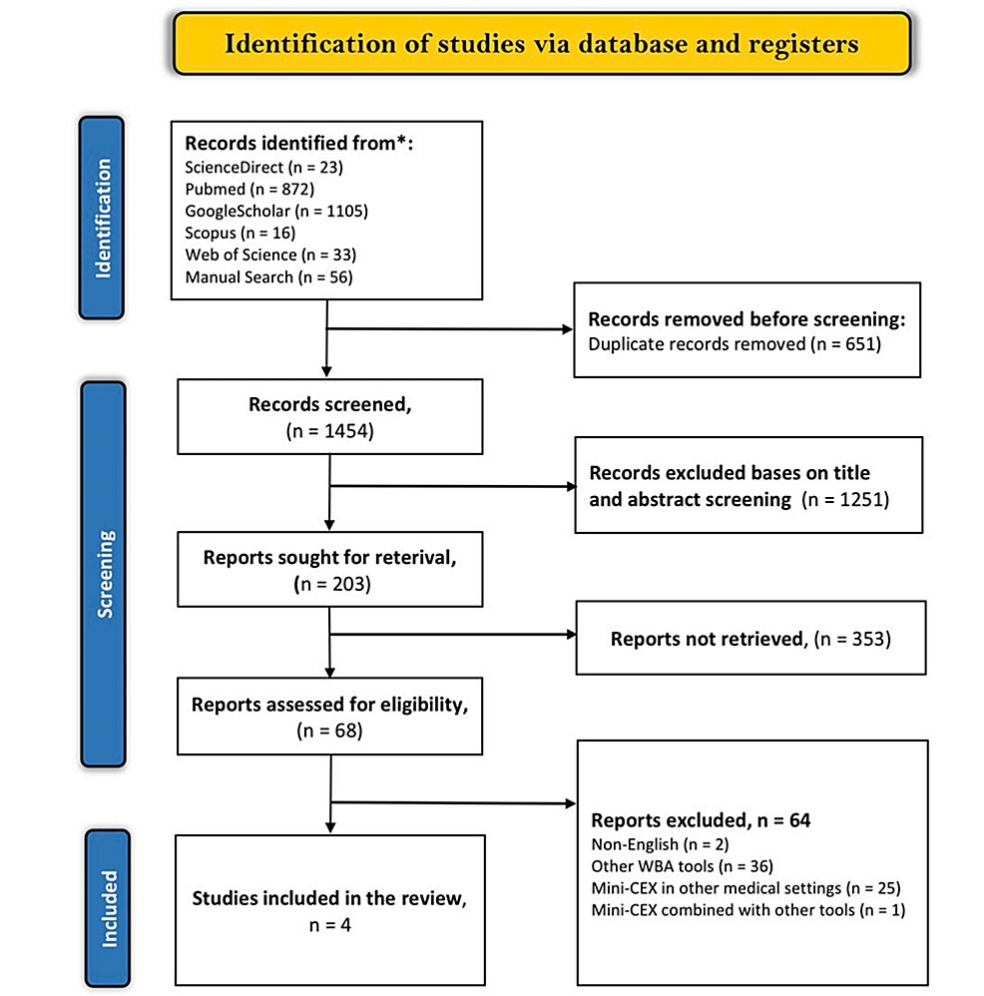
PRISMA flowchart illustrating the study selection procedure. PRISMA: Preferred Reporting Items for Systematic Reviews and Meta-Analyses

Our search for relevant articles using predefined search terms yielded 2,049 results. We also conducted a manual search which resulted in 56 additional articles. After screening for duplicates, we excluded 651 articles that were exact or close duplicates. The remaining articles were then screened based on their titles and abstracts, leading to the elimination of 1,251 articles. Of the 203 articles that remained, we were unable to retrieve 135 as they were only abstracts without full texts, systematic reviews, or study protocols. In the end, we included only four articles in our study and excluded the other 64 articles due to various reasons such as being published in different languages, evaluating WBA tools in different settings, evaluating mini-CEX in other medical settings, or evaluating mini-CEX combined with another assessment tool. The complete selection criteria can be found in the Preferred Reporting Items for Systematic Reviews and Meta-Analyses flow diagram shown in Figure 1.

Summary of the Study Characteristics

A summary of the study characteristics is presented in Table 1.

**Table 1:** Summary of the study characteristics. mini-CEX: mini-clinical evaluation exercise; EP: emergency physicians

Author ID	Study design	Country	Participants’ characteristics		Study period	Outcomes
			Residents/trainees (n)	Raters/faculty members (n)		
Chang et al., 2013 [7]	Retrospective study	Taiwan	183	57	17 months	The mean observation and feedback time by emergency physicians was 14.8 ± 8.8 and 11.0 ± 6.7 minutes, respectively. The faculty completion rate of mini-CEX in the first 12 months was 98.1%
Bashir et al., 2021 [8]	Questionnaire	Qatar	49	58	4 months	The faculty and residents’ completion rate of mini-CEX was 96%. The time taken to complete the mini-CEX assessment ranged between 10 and 20 minutes
Brazil et al., 2012 [9]	Cohort study	Australia	20	8	5 weeks	The median observation and feedback time of the mini-CEX encounter was 19 ± 7.47 and 10 ± 3.62 minutes, respectively. The faculty and residents’ completion rate of the mini-CEX survey was 87.5% and 95%, respectively). The overall satisfaction with mini-CEX as an assessment tool among the interns and assessors was high
Chang et al., 2017 [10]	Retrospective study	Taiwan	273	67	26 months	97.7% of EPs completed and provided feedback for mini-CEX. The overall observation and feedback time was 14.9 (10.9) and 11.1 (5.76) minutes, respectively

Quality Appraisal Results

After conducting a quality appraisal using the NOS, it was found that three studies had fair methodological quality, one had poor methodological quality, and none were of high quality. However, all the articles scored only two stars in the selection domain due to the absence of a control group. This made it impossible to identify the selection of non-exposed groups. Additionally, all studies were conducted in single centers, which meant that the ED trainees did not represent all trainees in other countries. In the study by Brazil et al., the comparability score was zero, as no confounders were adjusted for statistical analyses. Lastly, blinding the assessors to the outcomes was impossible, which prevented the studies from scoring total ratings in the outcome domain (Table 2).

**Table 2:** Methodological quality using the Newcastle-Ottawa scale.

Author ID	Selection (maximum 4)	Comparability (maximum 2)	Outcome (maximum 3)	Quality
Chang et al., 2013 [7]	2	1	2	Fair
Bashir et al., 2021 [8]	2	1	1	Fair
Brazil et al., 2021 [9]	2	0	1	Poor
Chang et al., 2017 [10]	2	2	2	Fair

Feasibility

**Figure 2:**
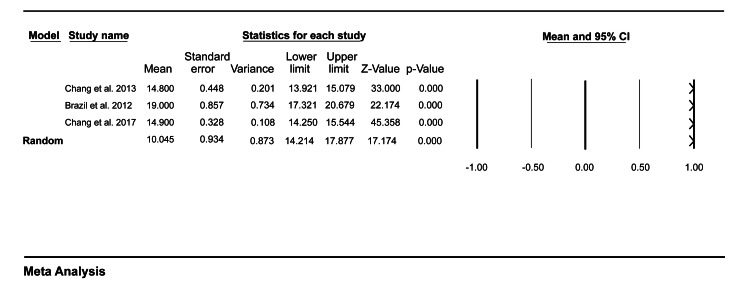
A forest plot showing the mean observation time for a single mini-CEX encounter. Chang et al. (2013) [7], Brazil et al. (2012) [9], Chang et al. (2017) [10]. mini-CEX: mini-clinical evaluation exercise

**Figure 3:**
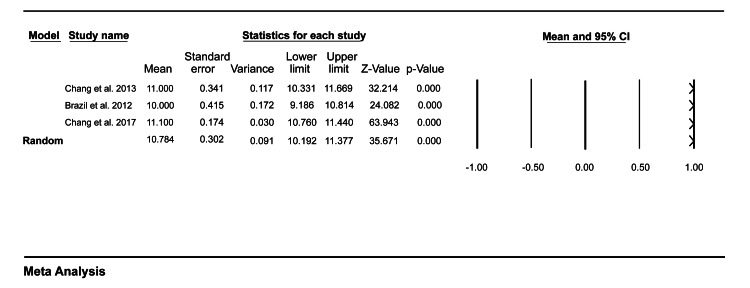
A forest plot showing the mean feedback time for a single mini-CEX encounter. Chang et al. (2013) [7], Brazil et al. (2012) [9], Chang et al. (2017) [10]. mini-CEX: mini-clinical evaluation exercise

Three studies included in our study reported outcomes related to feasibility. Data pooled from these studies showed that the mean time taken for observation of a single mini-CEX encounter was 16.05 (95% CI = 14.21-17.88) (Figure 2), and the mean time providing feedback to the medical trainees after the patient encounter in the ED was 10.78 (95% CI = 10.19-11.38) (Figure 3).

**Figure 4:**
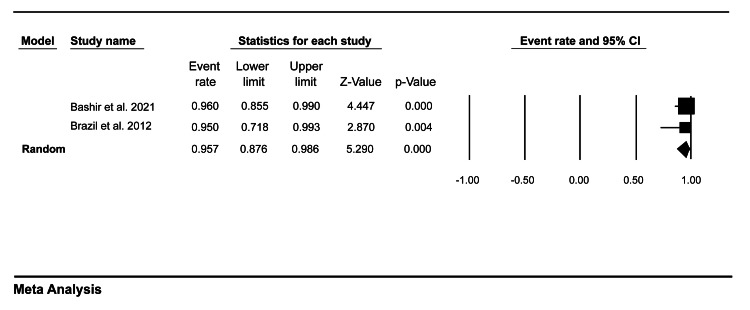
A forest plot showing the medical trainees’ completion rate of mini-CEX assessment. Bashir et al. (2021) [8], Brazil et al. (2012) [9]. mini-CEX: mini-clinical evaluation exercise

**Figure 5:**
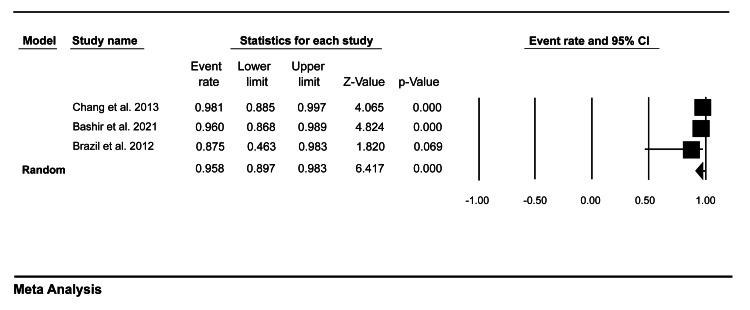
A forest plot showing the faculty members’ completion rate of mini-CEX assessment. Chang et al. (2013) [7], Bashir et al. (2021) [8], Brazil et al. (2012) [9]. mini-CEX: mini-clinical evaluation exercise

Moreover, our pooled analysis showed that the medical trainees’ completion rate was 95.7% (95% CI = 87.6-98.6) (Figure 4), while the faculty members’ completion rate was 95.8% (95% CI = 89.7-98.3) (Figure 5).

Acceptability

**Figure 6:**
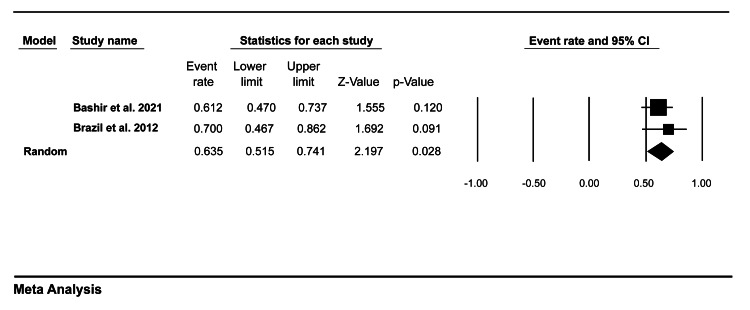
A forest plot showing medical trainees’ satisfaction with mini-CEX. Bashir et al. (2021) [8], Brazil et al. (2012) [9]. mini-CEX: mini-clinical evaluation exercise

**Figure 7:**
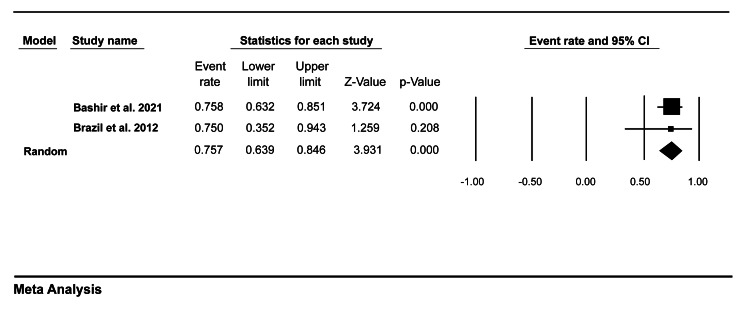
A forest plot showing faculty members’ satisfaction with mini-CEX. Bashir et al. (2021) [8], Brazil et al. (2012) [9]. mini-CEX: mini-clinical evaluation exercise

Only two studies in our review evaluated the assessor and trainees’ satisfaction with mini-CEX. Data pooled from these studies showed that 63.5% (95% CI = 51.5-74.1) of trainees tended to be satisfied with mini-CEX as an assessment tool (Figure 6), while 75.7% (95% CI = 63.9-84.6) of the faculty members were satisfied with mini-CEX as a formative tool (Figure 7).

Reliability

Due to a lack of adequate data, we could not perform a meta-analysis of reliability outcomes. Therefore, only descriptive analysis was conducted. According to Bashir and colleagues, most faculty members (70.6% (41/58)) agreed they could provide an appropriate time for mini-CEX during their clinical shifts. Moreover, the survey showed that most faculty members could complete the mini-CEX assessments during their clinical supervision shift. Therefore, mini-CEX might be a reliable assessment tool in the ED.

Discussion

The mini-CEX has been widely used to assess the clinical competence of medical students in various clerkships and residents undergoing different specialty training, such as internal medicine, cardiology, anesthesia, neurology, and dermatology [11-16]. However, its utility among residents and medical students in the ED is not widely documented. Therefore, this meta-analysis was undertaken to understand the feasibility, reliability, and acceptability of the mini-CEX utility in the ED.

Our meta-analysis has shown that the mean time required to observe a single mini-CEX encounter is 16.05 minutes, and the mean time to deliver feedback to the trainees is 10.78 minutes. These findings are validated by studies in different medical environments where the observation and feedback duration ranges from 10 to 25 minutes. For instance, Nair and colleagues reported that it took the assessors an average of 20 minutes to observe the international medical students as they interacted with the patients and 12 minutes to deliver feedback when using the mini-CEX [17]. Similarly, a 2021 randomized trial reported that mini-CEX assessments were completed in 25 minutes (i.e., 20 minutes for observation and 5-6 minutes for feedback) [18]. Furthermore, Norcini et al. (2003) evaluated mini-CEX as an assessment tool for internal medicine residents and found that the average time spent by the examiners to observe the residents as they interact with patients was 18 ± 12.1 minutes, and the mean time to provide feedback to the residents was 7.6 ± 5.3 minutes [19]. Considering the evidence from this literature and our meta-analysis, we can conclude that mini-CEX has a short observation and feedback time.

In addition, our meta-analysis has shown that the completion rate of mini-CEX forms among assessors and trainees was high (95.7% and 95.8%, respectively). These results are reinforced by the high mini-CEX completion rates in other medical settings. In a study of third-year medical students, the completion rate of personal digital assistants-based mini-CEX was 100% [20]. On the other hand, Gupta and colleagues reported a completion rate of only 50% [2]. This low completion was explained by faculty members and residents having busy schedules. However, due to various limitations, the evidence in that study cannot be used to preclude the use of mini-CEX in busy medical environments such as the ED. First, the study had a small number of encounters, meaning that the overall completion rate may have been influenced. Second, the study lacked diversity; therefore, it did not represent the entire population. Finally, the study was non-randomized, meaning that their statistical analyses were subject to error due to omission. This high completion rate reported in our study confirms that mini-CEX is a feasible assessment tool that can be integrated into training residents and medical students in the ED.

Our analysis has also shown that the mini-CEX has a high satisfaction level among assessors and trainees. This level of satisfaction is not surprising as the mini-CEX resembles the ordinary interaction between assessors and trainees in the clinical setting. Furthermore, the high satisfaction can be explained by the fact that some studies reported orientation programs where the trainees or faculty members were familiarized with mini-CEX before assessment. Bashir and colleagues reported that most of the faculty members had prior training on how to assess the trainees using the mini-CEX, meaning that their perception of the tool might have influenced their answers toward the acceptance of the assessment tool [8]. However, when interpreting the trainee satisfaction with mini-CEX, the tendency of the respondents to provide answers that are likely to impress their assessor should be taken into consideration.

Given that none of the studies included in our systematic review furnished statistical data for reliability measures, such as the generalizability coefficient and Cronbach’s alpha, we were unable to perform a meta-analysis on reliability. Nonetheless, a qualitative analysis of data from one of the studies has indicated the potential reliability of the mini-CEX for evaluating medical trainees in the ED. Nevertheless, this observation necessitates further high-quality studies to firmly establish whether the mini-CEX indeed qualifies as a reliable assessment tool in the ED.

Although the educational impact was beyond the scope of this review, the effect of mini-CEX on students’ learning is an integral part of its adoption in the ED. The educational impact in the included studies was indirectly analyzed through the feedback provided by the trainees. Bashir and colleagues reported that most emergency medicine residents agreed that the mini-CEX had improved their medical interviewing, physical examination, communication, professionalism, and organization skills [8]. Moreover, the study reported that most faculty members agreed that the mini-CEX had improved their attitude toward resident training. These findings imply that mini-CEX bears a favorable educational impact. However, there have been claims that the mini-CEX may not have any educational impact. For instance, in the study by Bashir et al. [8], two residents strongly disagreed that mini-CEX had enhanced their medical interviewing skills, and three strongly disagreed that it had improved their physical examination skills. These responses show that there is still a gap in the educational impact of the mini-CEX in the ED; therefore, future studies should emphasize on this outcome in the ED.

Cost-effectiveness is also considered an important factor for any assessment tool. Only one study in our review analyzed the cost of mini-CEX in the ED. Brazil et al. found that adding mini-CEX encounters was associated with increased cost [9]. In this study, the overall assessor time for a single senior medical officer was 36.5 hours which would represent 365 hours per year if the mini-CEX was carried out for each of the five intern rotations and at mid-term and end-term. Under these conditions, the direct and additional costs to the ED would be approximately $80,000 per year. Despite this study suggesting that the mini-CEX might be costly, it cannot be used to solely guide the preclusion of mini-CEX in the ED as it has other formative benefits. Moreover, the study had various limitations. First, only 20 interns were studied during the emergency medicine rotation, meaning that it was subject to a small sample bias. Second, removing 8 clinicians who would have contributed to the assessment process may also have influenced their outcomes. Finally, the study was carried out at the end of interns’ training when they had fewer deficiencies, meaning that the assessor time, which was correlated with the cost of mini-CEX, may have been influenced.

Factors that impact the utility of mini-CEX in the ED are also essential to its integration into emergency medicine training. The first factor that influences the mini-CEX is its format. Although our study did not differentiate the outcomes based on the format of mini-CEX, it usually exists as a paper-based or computer-based format. According to Chang and colleagues, the computer-based format improved data gathering more than the paper-based format [7]. The analysis in that study showed that out of the 475 paper-based mini-CEX in the first 12 months, incomplete data gathering was recorded in all seven aspects of clinical competency; however, no missing data were recorded when a computerized format was used in the next five months. Moreover, the study reported that the evaluators and postgraduate year one resident were more satisfied with the mini-CEX format, with a rating ranging from 5 to 9 (7.3 ± 0.8 and 7.9 ± 0.8, respectively). This is also evident in the study by Bashir et al. [8], where 61.2% of the emergency residents agreed that they were satisfied with the computer-based mini-CEX format. Similarly, Torre and colleagues, after comparing personal digital assistant (PDA) mini-CEX to the paper-based among medical students during their medicine clerkships, found that the residents’ satisfaction scores for the PDA formats were significantly higher than those of the paper format (7.2 ± 1.8 vs. 6.6 ± 1.7, respectively; p = 0.01) [21]. However, the faculty members seemed to be more satisfied with the paper format as opposed to the PDA format. These satisfaction differences suggest that electronic and paper-based mini-CEX should be used based on which format will likely meet the needs and preferences of instructors and learners.

In addition, the computer-based format of the mini-CEX seems to have various advantages that may prove important when utilized in busy environments such as the ED. First, this format usually allows the assessors to timely and effectively record the direct observation of trainees’ clinical skills, meaning that it saves time and effort associated with data gathering and recording, which is important in a busy ED. Second, collecting and analyzing data in a computer-based format is easy; hence, it can be used as a formative and longitudinal assessment tool. However, this does not mean that the paper-based format has no advantages. Using the paper-based format, the start-up cost for electronic devices and expenses associated with maintenance and development are minimized, meaning that the amount that would have been used to cater to these devices can be transferred to other developments in the ED. The paper format can also encourage the assessor to write more comments which can help with the documentation of feedback delivered to the medical trainees. However, due to an increase in technological advancements, more affordable portable electronic devices are now present, limiting the use of paper formats [22].

The other factor that may influence the utility of mini-CEX in the ED is the level of seniority. Chang and colleagues reported that junior faculty members were more satisfied with the computerized mini-CEX compared to the senior faculty members ((7.5 ± 0.8 vs. 6.9 ± 0.9, respectively, p < 0.001) [7]. Moreover, the study showed that junior faculty members tended to have longer observation and feedback time than senior faculty members. However, this difference was statistically insignificant. The high satisfaction level among the junior faculty members can be explained by the fact that they may be more familiar with the computerized assessment procedure than senior faculty members. Furthermore, research has shown that the level of seniority might influence the feedback provided by the assessors. Chang (2013) reported that the junior faculty member using the computer-based mini-CEX rated all the competencies (medical interviewing, physical examination, counseling skills, clinical judgment, organization skills, and professionalism) significantly higher than the senior faculty members [7]. Similarly, Torre et al. [20]. reported that residents rated the clinical competence of third-year medical students significantly higher than the faculty members based on PDA-based mini-CEX. This finding can be explained by the fact that the assessors’ own clinical skills might affect their rating of trainees, of which assessors with less experience/clinical skills tend to rate the trainees more highly than those with more experience.

As our meta-analysis has suggested that mini-CEX is a feasible and acceptable assessment tool within the ED, it is reasonable also to evaluate its validity. None of the studies included in our review provided any statistical data on the validity of the mini-CEX; however, Brazil et al. [9]. documented the perceived validity of mini-CEX. In their study, most respondents replied that the mini-CEX had a high face validity as a measure of intern performance. Moreover, most interns felt that the mini-CEX facilitated timely and effective feedback. The criterion validity of the mini-CEX has also been reported in previous studies. A 2013 systematic review analyzed the criterion validity of mini-CEX by comparing the trainees’ rating with other criterion measures and found that the effect size for overall clinical competence was “medium” (0.64; 95% CI = 0.48-0.77) [23]. The investigators of this review concluded that the mini-CEX has evidence of criterion validity after comparing it to other clinical skill achievements (e.g., oral and written examinations) or performance measures. Furthermore, a 2010 literature review by Hawkins and colleagues applied Kane’s framework to synthesize the evidence on the validity of mini-CEX and found that the mini-CEX was a valid assessment tool for clinical competence [24]. However, the authors argued that the weakest component of mini-CEX validity was based on its scoring component. They identified the following issues as the primary concerns of the scoring component: high inter-item correlation, rater selection and training, and leniency. Based on the findings from the literature, we can infer that mini-CEX is a valid assessment tool in different medical settings. However, more studies on its validity within busy EDs are still required to support this finding.

Although we have reported the reliability, feasibility, and acceptance of the mini-CEX as an assessment tool within the ED, its limitations should be considered. First, the direct observation using the mini-CEX seems to intimidate some medical trainees as they perform their routine clinical duties. According to Bashir and colleagues, some residents felt uncomfortable while being examined [8]. This sense of intimidation can be attributed to the natural fear of criticism, the nature of the assessors, and past interactions. Moreover, the uncomfortability can explain why the authors in that study were unable to attain a 100% satisfaction level with the mini-CEX as an assessment tool. Therefore, we recommend that students familiarize themselves with the mini-CEX before the assessment to enable them to overcome the discomfort. Second, due to a lack of time and a busy ED, it is difficult to complete all the mini-CEX forms. This was evident in our meta-analysis, where we have seen less than 100% completion rates among the assessors and trainees. Therefore, to overcome this challenge in busy EDs, we suggest that multiple mini-CEX components that allow students to self-identify opportunities to engage the faculty members are created. Finally, the assessment of the clinical competence of medical trainees is not unformed as it may be influenced by the assessor’s expectations and internal state [25]. Therefore, to overcome this limitation, we recommend that the faculty member undergo training before assessment.

Implications for practice and future research

Writing a clinical trial varies with respect to the audience it is intended [26]. From a practical standpoint, faculty members who want to use a feasible, acceptable, and reliable assessment tool within busy EDs can opt for the mini-CEX. However, it is worth noting that this tool has limitations that can hinder its applicability in the ED. Therefore, faculty members should consider using this tool with other WBA tools to improve its feasibility and acceptance. Moreover, there is a need for faculty members and trainees to familiarize themselves with the tool before assessment to eliminate intimidation and provide uniform feedback.

Our contemporary literature review has also identified some research gaps that future studies must address. First, there is limited research on the validity and reliability of the mini-CEX as an assessment tool within the ED; therefore, future articles should concentrate more on these factors. Second, none of the studies on mini-CEX have investigated how this assessment tool would influence trainees’ behavior as they interact with patients and how it would benefit patients. Therefore, future research should aim to address these concerns. Finally, the documentation of the mini-CEX as an assessment tool within the ED is still limited, and rigorous reporting is required to fully establish and support the findings reported in the current study.

Limitations

Like any other systematic review and meta-analysis, our study was also subject to various limitations to consider when interpreting the results. First, our research limited the search to articles published in the English language and studies with full articles only. This means that the data that would have been used to improve the statistical power of meta-analysis was eliminated based on these criteria. Second, we included studies with both the original and modified format of the mini-CEX in our analysis; therefore, our results may have been undermined. Moreover, we did not differentiate the data for computer-based and paper-based mini-CEX formats; thus, it is difficult to quantify how these formats influenced our meta-analysis results. Third, all the studies included in this review were non-randomized, and none had high methodological quality, meaning that all limitations that come with these studies were transferred to our analyses. Fourth, all studies included in this review were performed in single centers, which might not be representative of all centers worldwide, undermining our meta-analysis results. Finally, the reliability outcome reported in our study was inferred from only qualitative data in one study, which used questionnaires to gauge the opinions of faculty members and residents on the mini-CEX as an assessment tool. Therefore, it is difficult to determine whether the mini-CEX is a reliable assessment tool within the ED, and more studies are required to establish this finding.

## Conclusions

In summary, our meta-analysis indicates that the mini-CEX is a feasible and acceptable assessment tool within the ED. Nevertheless, the acceptability and feasibility of its application should be approached with careful consideration, given the presence of several concerns that could potentially limit its use as the sole assessment tool in the ED. Additionally, a qualitative analysis of data from one of the included studies suggests that the mini-CEX may also be a reliable assessment tool for medical trainees within the ED. However, a research gap persists regarding the reliability of this tool in the ED, and substantiating our assertion would require further high-quality studies.
